# Single-trial neurodynamics reveal N400 and P600 coupling in language comprehension

**DOI:** 10.1007/s11571-023-09983-7

**Published:** 2023-06-20

**Authors:** Christoph Aurnhammer, Matthew W. Crocker, Harm Brouwer

**Affiliations:** 1 Department of Language Science and Technology, Saarland University, Saarbrücken, Germany; 2 Department of Cognitive Science and Artificial Intelligence, Tilburg University, Tilburg, Netherlands

**Keywords:** ERPs, Language Comprehension, N400, P600, Neurolinguistics, Single-trial analysis

## Abstract

Theories of the electrophysiology of language comprehension are mostly informed by event-related potential effects observed between condition averages. We here argue that a dissociation between competing effect-level explanations of event-related potentials can be achieved by turning to predictions and analyses at the single-trial level. Specifically, we examine the single-trial dynamics in event-related potential data that exhibited a biphasic N400–P600 effect pattern. A group of multi-stream models can explain biphasic effects by positing that each individual trial should induce either an N400 increase or a P600 increase, but not both. An alternative, single-stream account, Retrieval-Integration theory, explicitly predicts that N400 amplitude and P600 amplitude should be correlated at the single-trial level. In order to investigate the single-trial dynamics of the N400 and the P600, we apply a regression-based technique in which we quantify the extent to which N400 amplitudes are predictive of the electroencephalogram in the P600 time window. Our findings suggest that, indeed, N400 amplitudes and P600 amplitudes are inversely correlated within-trial and, hence, the N400 effect and the P600 effect in biphasic data are driven by the same trials. Critically, we demonstrate that this finding also extends to data which exhibited only monophasic effects between conditions. In sum, the observation that the N400 is inversely correlated with the P600 on a by-trial basis supports a single stream view, such as Retrieval-Integration theory, and is difficult to reconcile with the processing mechanisms proposed by multi-stream models.

## Introduction

In electrophysiological research on language comprehension, the two most salient components of the event-related potential (ERP) are the N400 and the P600. While the N400 has traditionally been interpreted as an index of integrative-semantic processing (Brown and Hagoort 1993, 2000; Hagoort et al. 2004), the P600 was first discussed in relation to syntactic and structural processing (Hagoort et al. 1993; Osterhout and Holcomb 1992). Later studies challenged this functional distinction by eliciting “Semantic P600s” for manipulations in which thematic roles are reversed (“the javelin has the athletes thrown” relative to “the javelin was by the athletes thrown”, Hoeks et al. 2004, translated from Dutch) or grammatical inflections lead to implausible interpretations (“the hearty meal was devouring/devoured”, Kim and Osterhout 2005; see Brouwer et al. 2012; Bornkessel-Schlesewsky and Schlesewsky 2008; Kuperberg 2007 for reviews). Since then, theories of the electrophysiology of language processing are faced with the challenge to offer a unifying account of the mechanisms underlying the N400 and the P600 that can explain the sensitivities of both components.^1^ Specifically, Semantic P600 data gave rise to two alternative views on the language comprehension architecture: Multi-stream models (Kim and Osterhout 2005; van Herten et al. 2005; Kuperberg 2007; Bornkessel-Schlesewsky and Schlesewsky 2008; Kos et al. 2010; Michalon and Baggio 2019) and Retrieval-Integration theory (Brouwer et al. 2012, 2017), a single-stream model. Importantly, these theories are mostly informed by the binary presence and absence of N400 and P600 *effects* which are typically assessed by comparing mean amplitude across trials in a predefined time-window, such as 300–500 ms post-stimulus onset for the N400 and 600–1000 ms for the P600. A problem with this approach is that competing theoretical accounts may explain the same ERP data while assuming fundamentally different mechanisms. We here argue that important dissociations between competing effect-level explanations can be achieved by spelling out how different models envision the N400 and the P600 effect, observed between per-condition averages, to arise from language processing in single trials. Consequently, predictions derived from these single-trial level proposals can be investigated empirically in single-trial ERP data. In particular, we here demonstrate that by specifying predictions at the single-trial level, we can test two competing explanations of biphasic N400–P600 effects, offered by multi-stream models and Retrieval-Integration theory, respectively.

### Explaining N400 and P600 effects: multi-stream versus single-stream accounts

**Fig. 1:**
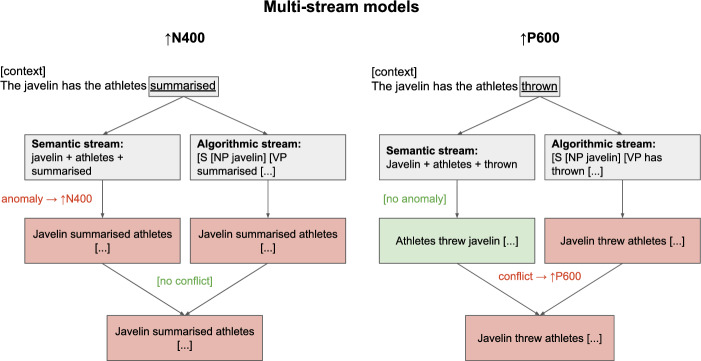
Schematic overview of the multi-stream explanation of N400 and P600 increases. Stimuli are examples from two conditions in Hoeks et al. (2004). Predicted N400 and P600 increases are specified relative to the baseline condition (“the javelin was by the athletes thrown”). Hoeks et al. (2004) found an N400 effect and a P600 effect for “The javelin has the athletes summarised” and a P600 effect for “The javelin has the athletes thrown”, relative to baseline. All examples transliterated from Dutch

Multi-stream models were developed in order to reconcile the integration view of the N400 with the absence of N400 effects and the presence of P600 effects in Semantic P600 studies by postulating that language processing makes use of two processing streams (but see Kuperberg 2007, for an account with three streams). While the precise conceptualisation of the different processing streams varies across multi-stream models, they share several critical elements: Typically, a *semantic* processing stream employs a plausibility heuristic that constructs an utterance meaning representation based on the content words of the input, while ignoring syntactic constraints (see Kim and Osterhout 2005; van Herten et al. 2005; Kuperberg 2007; Bornkessel-Schlesewsky and Schlesewsky 2008; Kos et al. 2010, for a more detailed discussion and see Rabovsky et al. 2018; Ryskin et al. 2021; Li and Ettinger 2023; Michalon and Baggio 2019, for more recent models with a similar processing mechanisms). Critically, for some experimental conditions, no increase in N400 amplitude is taken to occur if the content words make a plausible alternative interpretation available, e.g., by ignoring word order in role-reversed input and assuming the most probable interpretation instead (e.g., interpreting “the javelin has the athletes thrown” as “the javelin was by the athletes thrown”; Hoeks et al. 2004). The *algorithmic* processing stream, however, does adhere to morphological, syntactic, and structural constraints and detects the anomaly in the input (Kim and Osterhout 2005; van Herten et al. 2005; Kuperberg 2007; Bornkessel-Schlesewsky and Schlesewsky 2008; Kos et al. 2010, see also Rabovsky and McClelland 2020; Ryskin et al. 2021; Li and Ettinger 2023; Michalon and Baggio 2019, for more recent examples). According to multi-stream models, it is the conflict between the analyses generated by the semantic (the athletes threw the javelin) and the algorithmic processing stream (the javelin threw the athletes) that gives rise to the increase in P600 amplitude (see Fig. 1, right). However, if the anomalous condition does not make a semantically attractive alternative interpretation available (“The javelin has the athletes summarised”), an increase in N400 amplitude is predicted to be produced by the semantic stream, and the two streams agree in their analyses. Hence, there is no conflict and no increase in P600 amplitude is predicted—contra to the findings of Hoeks et al. (2004; see Fig. 1, left).

**Fig. 2:**
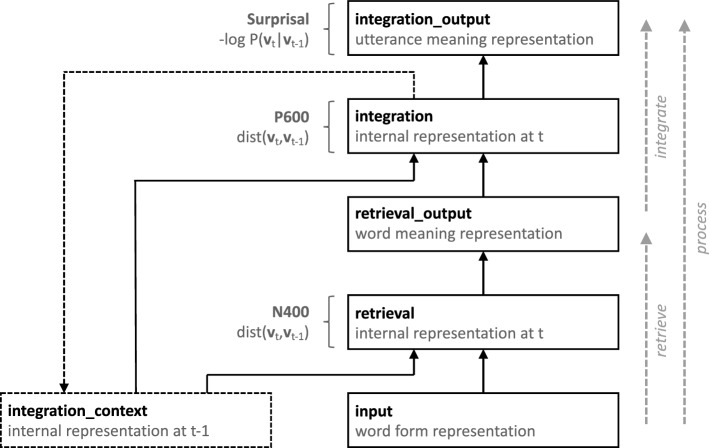
Schematic architecture of the neurocomputational instantiation of Retrieval-Integration theory, implementing word-by-word language processing and the linkage of retrieval to the N400 and integration to the P600. For full detail on model implementation see Brouwer et al. (2021)

An alternative, single-stream, account of the N400 and the P600 is Retrieval-Integration (RI) theory (Brouwer et al. 2012, 2017). On the RI account, the N400 is taken to index lexical retrieval (Kutas and Federmeier 2000; Lau et al. 2008, 2009; van Berkum 2009, 2010), i.e., the retrieval of word meaning from long-term memory, and the P600 is posited to index integration, the updating of an utterance meaning representation with the meaning of the current word. RI theory posits that the N400 and the P600 are elicited by every word and that their amplitudes are continuous indices of retrieval effort (N400) and integration effort (P600), respectively. Fig. 2 depicts a schematic of the computational instantiation of RI theory proposed by Brouwer et al. 2021). In this model, the amplitudes of the N400 component and the P600 component are taken to be proportional to the word-by-word change in the **retrieval** and **integration** layers, respectively. According to this model, no N400 *effect* between conditions is observed, if conditions facilitate retrieval equally, and no P600 *effect* between conditions is observed if integration is equally effortful in the conditions. Indeed, this explanation is consistent with the absence of an N400 effect for the sentence “The javelin has the athletes thrown” relative to the baseline “the javelin was by the athletes thrown” (Hoeks et al. 2004), as the target word is similarly associated to the context in both conditions. The P600 effect is explained by the implausibility of the role-reversed sentence relative to the control sentence.

### Dissociating effect-level explanations at the single-trial level

**Fig. 3:**
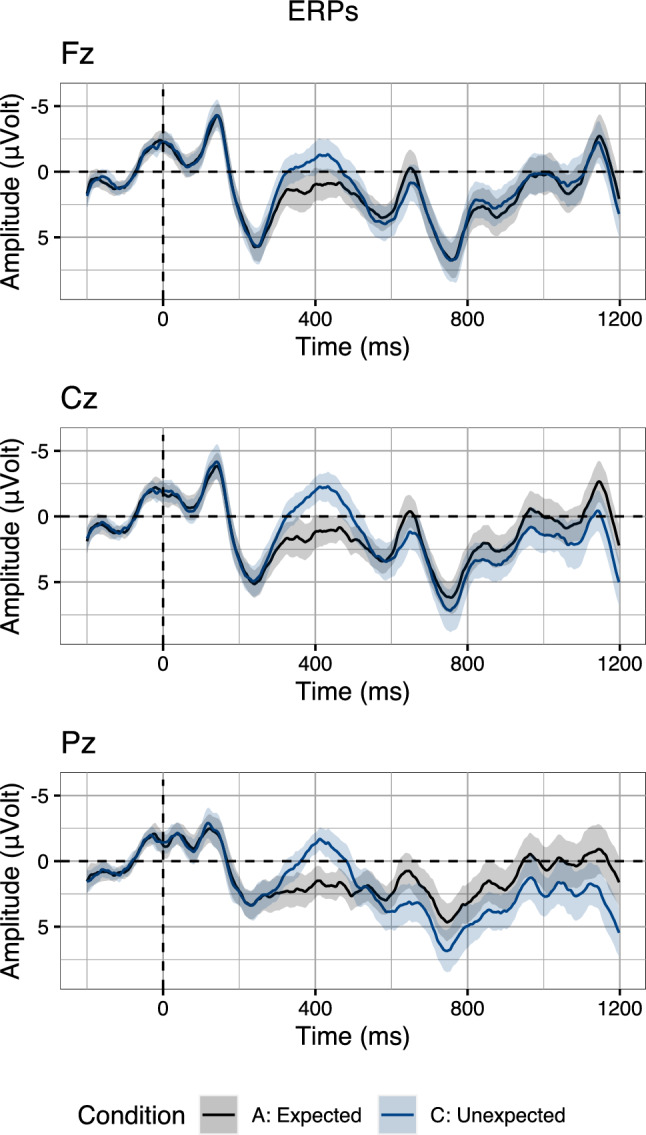
Grand-average ERPs on three midline electrodes (Fz, Cz, Pz) for two conditions of Aurnhammer et al. (2021) that manipulated target word expectancy. Waveforms were averaged per-condition from the per-subject per-condition averages. Error ribbons indicate confidence intervals based on standard errors computed across subjects

Multi-stream models were strongly motivated by the monophasic P600 effects and monophasic N400 effects observed in Semantic P600 studies. However, several condition contrasts in these studies also elicited biphasic effects (e.g., Hoeks et al. 2004). Further, recent studies demonstrated that component overlap between the N400 and the P600 can result in the attenuation or absence of P600 effects (Brouwer et al. 2021; Delogu et al. 2021, 2019). Indeed, consulting the empirical evidence, it is striking that language comprehension ERP experiments manipulating semantic congruency (e.g., “He spread the warm bread with socks/butter”, Kutas and Hillyard 1980) often elicit *biphasic* ERP responses consisting of both an N400 effect and a P600 effect relative to baseline (see Van Petten and Luka 2012, for an overview). For instance, a recent experiment (Aurnhammer et al. 2021) manipulated the expectancy of the target word (“Yesterday, sharpened the lumberjack [...] the axe” vs. “Yesterday ate the lumberjack [...] the axe”; transliterated from German, see Table 1). The Unexpected condition elicited both a more negative N400 amplitude and a more positive P600 amplitude, relative to the Expected baseline condition (Fig. 3).

**Table 1:** Example item, showing the expectancy manipulation of Aurnhammer et al. (2021), achieved by violating the selectional restrictions of the main verb (“sharpened/ate”)

Expected	Yesterday sharpened the lumberjack [...] the axe and.
Unexpected	Yesterday ate the lumberjack [...] the axe and.

Stimuli are transliterated from German, preserving word order. Target words were underlined for this table. The original design also manipulated lexical association of the target word to the words in an adverbial clause preceding the target word (“before he the [wood stacked/movie watched]”). In the two conditions shown here, the adverbial clauses are identical, associated, and omitted in the table

Semantic P600 studies employed experimental designs that maximised the presence/absence of semantic attraction, which on multi-stream accounts should determine the presence/absence of P600/N400 increases. Because of this, the biphasic effect patterns observed in some Semantic P600 studies (e.g., Hoeks et al. 2004) have been discussed as difficult to reconcile with multi-stream models (Brouwer et al. 2012). However, in cases of canonical semantic incongruities, one possible multi-stream explanation of biphasic effects could be that the N400 and P600 effect observed in the *averages* derive from trial-specific N400-only and P600-only elicitations. That is, in the case of canonical semantic incongruities, it is not always clear whether all experimental items exclude the presence of a semantically attractive alternative interpretation for the incongruent items, especially if a broad notion of global semantic attraction is adopted (see Kuperberg 2007; Bornkessel-Schlesewsky and Schlesewsky 2008; Aurnhammer et al. 2023, for discussion). Hence, it would be conceivable that for one subset of the unexpected trials there was no semantically attractive alternative interpretation and the unexpected target word was detected in the semantic stream, which resulted in an N400 increase. However, for the remaining unexpected trials, there may have been semantic attraction, and the input may have been judged as plausible in the semantic stream, leading to no N400 increase. In the algorithmic stream, these trials would however result in an implausible analysis and the conflict between the analyses of the semantic and the algorithmic stream should induce a P600 increase (see Fig. 1). Averaging over these two subsets of anomalous trials may result in the biphasic condition contrast in which the average N400 is more negative and the average P600 is more positive in the incongruent condition than in the baseline.

Crucially, and in contrast to multi-stream accounts, RI theory predicts both an N400 and a P600 increase on the same trials: On RI theory, both the mapping of word forms to word meanings (retrieval) and the mapping of word meanings into an updated utterance meaning representation (integration) are constrained by utterance context—the utterance meaning constructed so far (see Fig. 2). Hence, for the processing of a single word, the single-stream architecture makes a fundamental prediction: Due to the shared dependency of retrieval and integration on the utterance meaning constructed so far, words that are more effortful to retrieve should also be more effortful to integrate. Consequently, N400 amplitude and P600 amplitude should be inversely correlated. This prediction is supported by quantitative model estimates generated by the computational instantiation of RI theory (Brouwer et al. 2021) for a recent ERP study by Delogu et al. (2019). Comparing the N400 and P600 estimates generated by this model for all words in the stimuli (i.e., not just the target words), we indeed find a negative correlation ($r = -0.62$). The model estimates thus confirm the prediction of RI theory that words with a more negative N400 amplitude should generally also induce a more positive P600 amplitude.

Thus, multi-stream models and RI theory account for biphasic effect patterns, by assuming very different processing architectures that, critically, make opposing predictions for the modulation of the N400 and the P600 within-trial: On multi-stream accounts, the presence of a N400 increase for an anomalous trial predicts the absence of a P600 increase and, vice versa, the absence of an N400 increase for an anomalous trial predicts the presence of a P600 increase. In contrast, RI theory predicts that N400 amplitude and P600 amplitude should be inversely correlated in that more negative N400 amplitudes should co-occur with more positive P600 amplitudes. To investigate the single-trial dynamics of the N400 and the P600, we re-analyse the ERP data presented by Aurnhammer et al. (2021). While the full experiment crossed expectancy with lexical association, we here focus only on the expectancy manipulation. Indeed, both multi-stream models and RI theory offer a possible explanation for this expectancy manipulation at the effect-level. We will here examine, however, whether they can also account for the data at the single-trial level.

Quantitatively, the prediction of multi-stream models can be expressed, slightly unintuitively, by a *positive* correlation between N400 amplitude and P600 amplitude relative to the grand-average of two conditions with a biphasic effect (Fig. 3). That is, on multi-stream accounts, a trial that results in an increase in N400 amplitude, should not result in an increase in P600 amplitude. As a consequence, P600 amplitude should be more negative than the grand-average in this case. Conversely, a trial that results in an increase in P600 amplitude, should not result in an increase in N400 amplitude. Hence, in this case, N400 amplitude should be more positive than the grand-average. Taken together, this predicted pattern thus results in a *positive* correlation between N400 amplitude and P600 amplitude at the single-trial level: If P600 amplitude becomes more positive, N400 amplitude should not diverge from baseline, and hence be more positive than the grand-average, and vice versa.

The prediction of RI theory, on the other hand, can be expressed by a *negative* correlation between N400 and P600 amplitudes at the single-trial level. That is, RI theory assumes that both retrieval and integration are expectation-based processes: The expectations about upcoming word meaning (retrieval) and utterance meaning (integration) both derive from the utterance meaning representation constructed so far. Hence, it is due to this shared dependency on the unfolding utterance meaning representation that RI theory predicts unexpected words to generally—on a by-trial basis—be more difficult to retrieve and more difficult to integrate. This results in the prediction that there is a negative correlation between N400 amplitude and P600 amplitude, because more negative N400 amplitudes should co-occur with more positive P600 amplitudes, and conversely, more positive N400 amplitudes with more negative P600 amplitudes. After the analysis of ERP data that elicited a biphasic effect (Aurnhammer et al. 2021), we also test the generalisability of the proposed single-trial neurodynamics to ERP data that elicited only monophasic effects between conditions (Delogu et al. 2019).

## Method

Both multi-stream models and RI theory can explain condition contrasts resulting in a biphasic N400–P600 effect. In order to investigate whether, and if so, how the N400 amplitudes of single trials correlate with the P600 amplitudes of the same trials, we re-analyse the data in the Expected and Unexpected conditions of Aurnhammer et al. (2021; Table 1, Fig. 3). Our analyses focus on three midline electrodes, as we did not observe hemispheric differences in the topography of the N400 effect and the P600 effect. In the electroencephalography (EEG) experiment, 120 items were presented to 40 participants. After artefact rejection, 2027 trials remained in the subset of the Expected and Unexpected conditions. Sentences were presented using rapid serial visual presentation, whereby individual words were presented centrally on the screen for 350 ms with a 150 ms inter-stimulus interval. After presentation of each sentence, participants were instructed to provide a binary plausibility judgement. The EEG was re-referenced offline to the average of the left and right mastoid electrodes and band-pass filtered between 0.01 and 30 Hz. Data were baseline corrected using a 200 ms pre-stimulus interval. For full detail on experimental design, electrophysiological recording and processing, refer to Aurnhammer et al. (2021). All data and code required to reproduce the analyses is publicly available.^2^

### Towards single-trial dynamics: naive binning-based approach

**Fig. 4:**
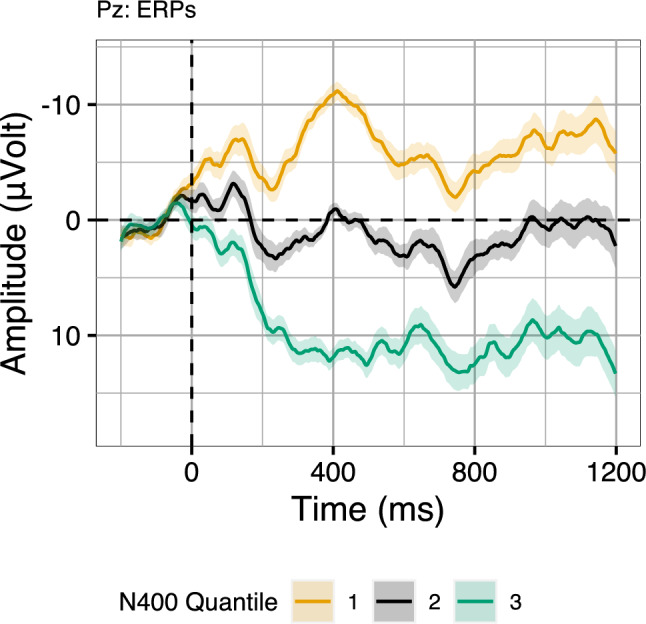
EEG signals binned by N400 averages (300–500 ms) in the Expected and Unexpected conditions of Aurnhammer et al. (2021) on electrode Pz. Error ribbons indicate confidence intervals based on standard errors computed across quantiles

An initial approach to investigate the interrelation of N400 and P600 amplitudes would be to compare their raw amplitudes. Computing their correlation, we find that in fact, single-trial N400 amplitudes (300–500 ms) and P600 amplitudes (600–1000 ms) are positively correlated ($r = 0.67$; correlation computed for electrode Pz, where both the N400 effect and P600 effect were maximal; see Aurnhammer et al. 2021, for topographic maps). That is, trials with more negative N400 amplitude also appear to exhibit more negative P600 amplitudes and vice versa. At face value, this supports the multi-stream explanation rather than RI theory. To validate whether this positive correlation between the amplitudes in the two time-windows is indeed specific to the ERP components of interest, we compute per-trial averages in the N400 time-window in order to split the data into three equal sized bins. This binning is then applied to visualise the entire waveforms. With regard to the predictions, we then examine whether the bins derived from the N400 time-window also induce an ordering in the P600 time-window. The resulting bins for electrode Pz, on which both the N400 effect and the P600 effect were maximal in the original experiment, are displayed in Fig. 4.

While some of the typical peaks and troughs of visually elicited language ERPs are visible in the bins, it is striking that the bin-averaged waveforms diverge immediately after stimulus onset, i.e., the point from which baseline correction takes effect. This immediate divergence of the bins before the N400 time-window casts doubt on the idea that the correlation between the single-trial averages in the N400 and the P600 time-window, as visualised by the three bins, captures (only) systematic N400 variability.

**Fig. 5:**
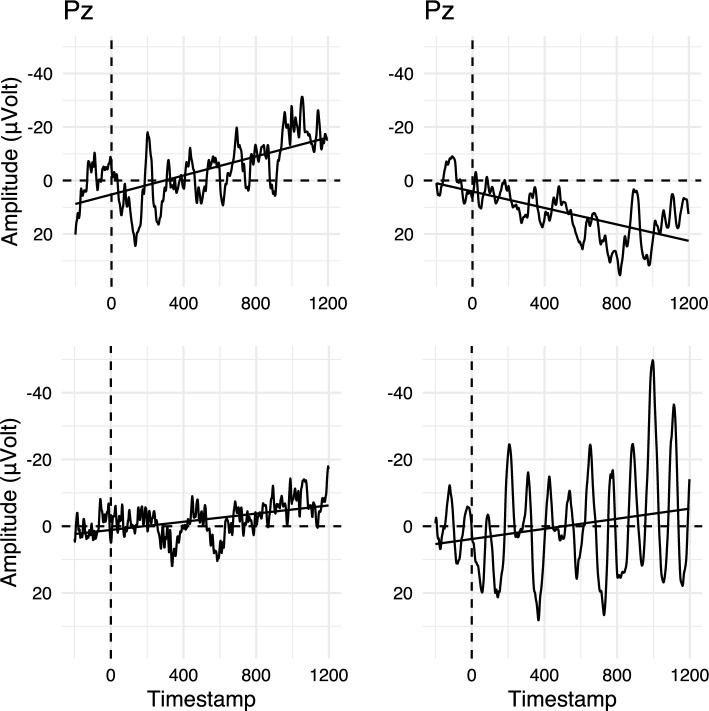
Four randomly selected single-trial waveforms from the Expected and Unexpected conditions in Aurnhammer et al. (2021). Regression lines indicate voltage trends over time

To understand how these bins arise, it is useful to consider the kinds of noise and variability present in single-trial EEG data (Fig. 5). Overall, single-trial EEG signals are characterised by a low signal-to-noise ratio. Unsystematic variation—i.e., variability not elicited by the stimulus—comes in the form of random noise, periodic signals, such as alpha waves, or as monotonous voltage drifts that are becoming more negative or positive over time (highlighted by regression lines in Fig. 5). Single-trial N400 time-window averages will thus be driven by unsystematic variability (such as voltage drifts) to a much larger extent than by the underlying N400 amplitude within this trial. The averaging of ERP signals per conditions and/or per subject removes drifts from average ERPs if they are occurring randomly, that is, if they do not systematically co-occur with specific conditions and/or subjects. When computing three N400 bins based on the “raw” N400 time-window average (Fig. 4), we are however grouping the data based on a property of the signal itself and hence the resulting bins are not independent from the noise. Thus, the bins may be more strongly driven by the overall amplitude magnitude of the signals than by true N400 amplitude. This explanation is supported not only by the immediate divergence of the bin-averaged waveforms after stimulus onset but also by the overall magnitude of the highest and lowest bin (compare to the condition averages of Fig. 3). Importantly, some kinds of noise, such as voltage drifts, are correlated in two consecutive time-windows, which could thus alternatively explain a positive correlation of N400 amplitudes and P600 amplitudes. Hence, in order to group the EEG data based on some characteristic of the signal itself, such as the size of the N400 in a single-trial, or to compute correlations between consecutive time-windows, it is necessary to separate voltage drifts from the systematic N400 modulations.

**Fig. 6:**
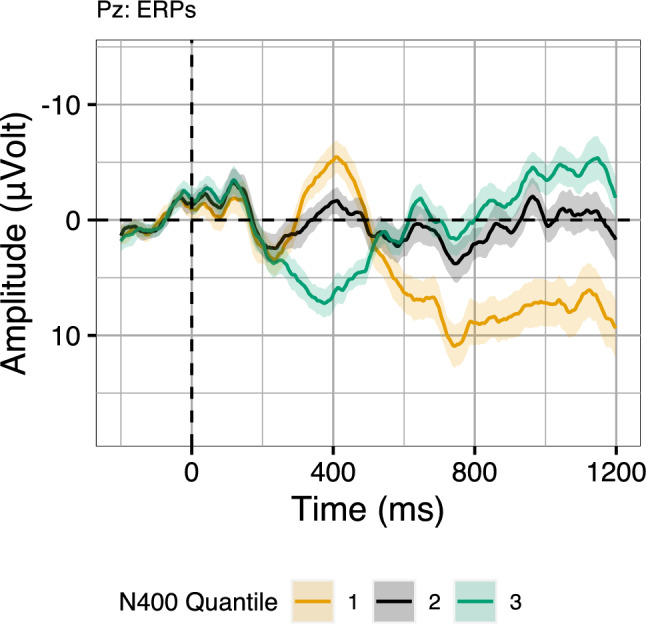
EEG signals grouped by bins obtained by subtracting average Segment voltage (0–1200 ms) from average N400 voltage (300–500 ms) in the Expected and Unexpected conditions of Aurnhammer et al. (2021). Error ribbons indicate confidence intervals based on standard errors computed across quantiles

A naive approach to remove the voltage drift from the binning would be to compute the average of the N400 time-window and subtract from it the average voltage on that trial computed from 0 to 1200 ms post-stimulus onset (cf. the traditional procedure for applying baseline correction). Crucially though, these “average N400minus average Segment” voltages are *only* used to arrive at the bins, which are then used to visualise the *unaltered* data as bin-averaged waveforms (making this approach different from baseline correction). That is, we do not alter the displayed data in any way: the subtraction procedure only affects the assignment of trials to bins. Interestingly, if we apply the subtraction-based binning, the resulting average waveforms better resemble typical condition-average ERP waveforms (Fig. 6). Most strikingly, the waveforms do not diverge immediately after stimulus onset. Rather, it is only around 300 ms (the beginning of the N400 time-window) that the waveforms start to diverge, suggesting that the subtraction procedure may indeed have recovered aspects of systematic N400 variability in the single-trial N400 voltages, while removing random voltage drifts. Crucially, moving to the P600 time-window (from around 600 ms post-stimulus onset), the ordering of the N400 bins in fact flips. That is, according the to subtraction-based bins, the more negative the N400 amplitude, the more positive is P600 amplitude. The validity of the obtained binning is strengthened by the morphology of the resulting waveforms, which, compared to Fig. 4, suggest clearer N400 components and P600 components with more typical peaks and latencies, as well as no large differences before them (pre 300 ms). This subtraction-based binning approach can also be related back to quantitative correlations, by computing the partial correlation between N400 amplitudes and P600 amplitudes that accounts for their correlation to Segment voltage: While the raw correlation between N400 amplitude and P600 amplitude was positive, the partial correlation that factors out Segment voltage is negative ($r = -0.49$, correlation computed for electrode Pz).^3^ In sum, the naive subtraction based binning approach suggests that the bin with the largest N400 amplitudes in fact also includes the largest P600s, indicating a negative correlation on a by-trial basis between the two components.

Hence, if voltage drifts are accounted for, the correlation as well as the binning, which are both based on the single-trial EEG data, are incompatible with the explanation of biphasic data that we articulated for multi-stream accounts: The architecture of most multi-stream models suggests that trials which induce more negative N400 amplitudes do not trigger a P600 increase and vice versa. The language processing architecture proposed by Retrieval-Integration theory directly predicts the obtained pattern, because retrieval effort (N400) and integration effort (P600) should be correlated negatively at the target word. While these results indeed form initial support for a coupling of the N400 and the P600 at the single-trial level, this naive approach still suffers from shortcomings.

### Towards single-trial dynamics: regression-based approach

The subtraction used in the binning process is rather crude and applies the same amount of subtraction ($\text {N400} - 1 * \text {Segment}$) to all time-steps. This is inadequate because voltage drifts tend to be directed (see Fig. 5), i.e., they become more negative or positive over time (see also Hennighausen et al. 1993). Due to this directedness, it would be desirable to apply a variable amount of drift correction across time. Ideally, the optimal amount of voltage correction should be derived from the data itself at each time sample. This can be achieved straightforwardly by casting the research question into the perspective of rERPs (Smith and Kutas 2015), a regression based ERP analysis technique. At the core of the rERP technique lies the observation that fitting a series of intercept-only regression models—one for each subject at each time sample—is mathematically equivalent to computing a grand-average ERP waveform from per-subject average waveforms, meaning that “all ERPs *are* rERPs” (Smith and Kutas 2015, p. 158). Building on this, more predictors can be added to the regression equations in order to model the variability around the mean, and across time samples in the EEG signal. For instance, the original data from both conditions could be modelled using a continuous predictor, such as cloze probability (see the analyses in Aurnhammer et al. 2021). Here, however, we are interested in explaining the EEG signals recorded from each subject and at each time-step as a function of that signal itself in order to determine a possible coupling of N400 and P600 amplitude.

Hence, our rERP models^4^ include the average N400 voltage (300–500 ms) and the average Segment voltage (0–1200 ms) as trial-level predictors (see Alday 2019, for a similar approach to applying baseline correction). We apply the analysis method to three midline electrodes and compute separate N400 and Segment predictors for each electrode. Predictors are z-standardised and inverted. While the inverting results in positive correlations to be expressed by negative coefficients on the N400 predictor (and vice versa), it will aid intuitive ERP-like visualisation of the resulting model coefficients. We arrive at a set of models of the following form:

1 $$\begin{aligned} {\hat{y}}_{st} = \beta {0}_{st} + \beta {1}_{st} * \text {N400}_{st} + \beta {2}_{st} * \text {Segment}_{st} \end{aligned}$$

These regression equations compute estimated data ${\hat{y}}$ for each subject *s* and time sample *t*. The intercept term $\beta 0$ will equal the average of the data for the current selection of subject and time sample. As both other predictor terms, N400 and Segment, are computed per-trial, they allow us to capture any auto-correlations present in the signal. Specifically, the Segment voltage predictor fitted by coefficient $\beta 2$ will capture the extent to which the EEG signal, across time samples, is explainable by overall segment magnitude. Seeing that voltage drifts tend to be directed, becoming more positive or negative over time (Fig. 5), we expect that the Segment voltage coefficient should increase over time. The N400 predictor, on the other hand, captures the extent to which N400 amplitude explains variability in the EEG signal, over and above what is explained by the Segment predictor. The combined presence of both predictors in the models effectively leads to a variable weighting of both predictors over time, which is expressed in the magnitude of the coefficients. Hence, our rERP analysis should be superior to the invariable subtraction-based approach. We expect the N400 average predictor, fitted by coefficient $\beta 1$, to be a very good predictor for the N400 time-window itself. Our prediction about the N400–P600 single-trial dynamics is addressed by inspecting the coefficients of the N400 predictors in the P600 time-window (600–1000 ms). If a positive correlation exists between N400 and P600 amplitude, as predicted by multi-stream models, the N400 coefficient should extend its trend from the N400 time-window into the P600 time-window. If, on the other hand, N400 and P600 amplitude are inversely correlated, the N400 coefficient should flip sign when moving from the N400 to the P600 time-window and predict more positive amplitudes from around 500 milliseconds post-stimulus onset.

#### Single-trial dynamics across conditions

**Fig. 7:**
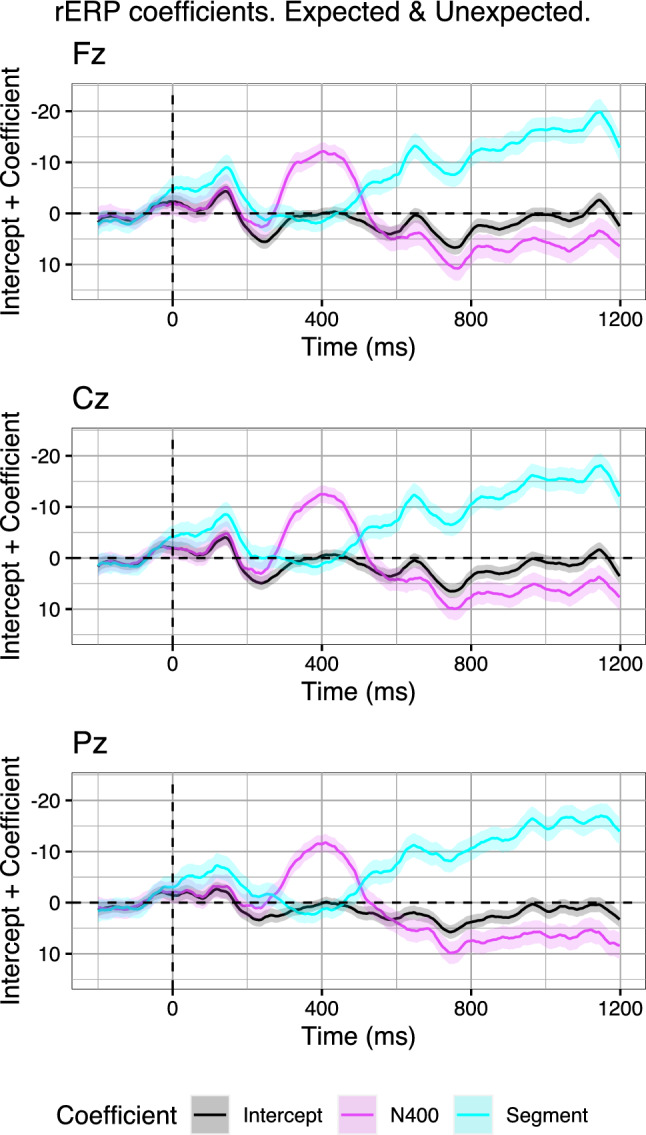
Model coefficients (added to their intercept) across time on three midline electrodes (Fz, Cz, Pz) for regression models fitted on two conditions of Aurnhammer et al. (2021). Coefficients express the extent to which single trial N400 amplitude (averaged from 300 to 500 ms) and Segment amplitude (averaged from 0 to 1200 ms) explain the EEG signal across time. Error ribbons indicate standard errors on the coefficients

The resulting coefficient graph (Fig. 7; coefficients are added to the intercept) demonstrates that the Segment predictor becomes active—relative to the intercept—immediately after stimulus onset and, indeed, the coefficient increases over time, suggesting that it captures monotonically increasing and decreasing voltage drifts. Critically, the Segment coefficient drops back to the intercept during the N400 time-window, indicating no contribution to explaining the signal. This is simply because the N400 predictor captures both systematic and random variability in its own time-window very well. After the N400 time-window, the previous trend of the Segment predictor continues. In sum, the coefficients of the Segment predictor indicate that trials that are more negative overall tend to become more negative over the course of the segment and those that are more positive overall become more positive over the course of the segment. The coefficient for the N400 predictor, on the other hand, indicates only a small contribution prior to the N400 time-window and the directionality of the coefficient indicates that more negative voltages in the N400 time-window predict more negative voltages prior to the N400 time-window. In the N400 time-window itself, the coefficient of the N400 predictor increases in magnitude. Here, the N400 predictor presumably models both the systematic N400 variability and the Segment drifts present in this time-window (cf. the “raw” N400 bins above, Fig. 4).

The critical aspect of the rERP analysis is the behaviour of the N400 predictor in the P600 time-window (600–1000 ms). Indeed, in the P600 time-window, the coefficient of the N400 predictor changes sign, indicating that trials that were more negative in the N400 time-window are predicted to become more positive in the P600 time-window. Importantly, due the presence of the Segment predictor, voltagedrift-related variability in the N400 predictor is factored out when determining the latter’s best-fit coefficient—at least to the extent to which the Segment predictor accounts for the drift related variability. In sum, the rERP analyses, in which Segment correction is optimised for each subject and time sample, support the initial results derived from the naive subtraction-based binning approach: Trials with more negative N400 amplitudes also induce more positive P600 amplitudes.

**Fig. 8:**
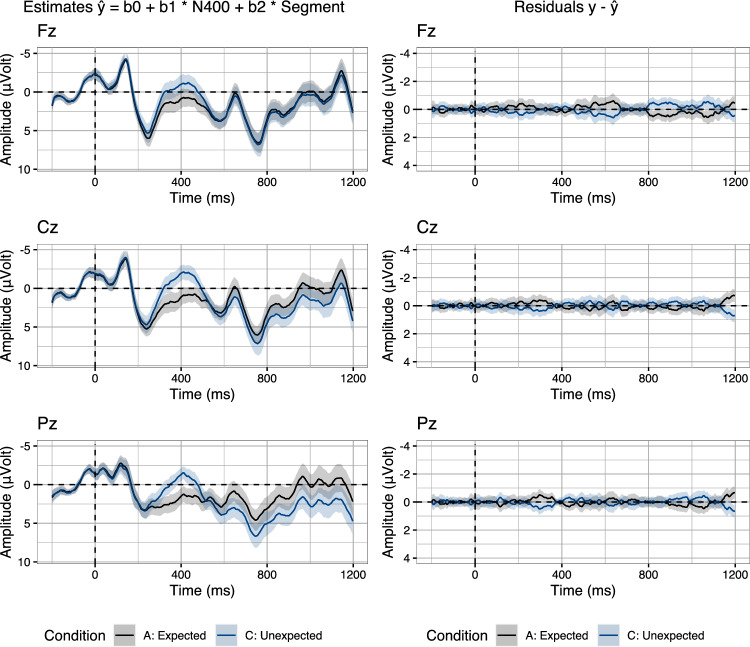
Forward estimates (left) and residual error (right) on three midline electrodes (Fz, Cz, Pz) from a set of regression models fitted using Eq. 1. Estimates and residuals were split per condition. Error ribbons indicate confidence intervals based on standard errors computed across subjects

Crucially, the rERP approach still suffers from one shortcoming: It is currently not possible to quantify the extent to which the predictors—which are derived from the signal itself—pick up on N400–P600 dynamics or on noise that is correlated across time windows. In order to clarify this issue, we return to the traditional approach of removing randomness in EEG signals, which is the averaging of noisy single-trial EEG recordings to average ERPs. The intuition is that noise which randomly occurs with the grouping factor used for averaging (e.g., conditions) will be removed in the average ERPs. Inspired by this traditional approach, we evaluate our rERP analysis by measuring the extent to which the N400 and the Segment predictor are able to recover the two conditions underlying the current data (see Fig. 3, Table 1). In order to evaluate the rERP models against the two conditions, we use the regression coefficients to compute the estimates (${\hat{y}}$ in Eq. 1) for the entire data set. We then group the estimates by the original two conditions (Fig. 8, left), in order to determine the extent to which the estimates reproduce the biphasic N400–P600 effect pattern. Indeed, compared to the original two conditions (Fig. 3), the estimated data appear to capture both the N400 effect and, more importantly, the P600 effect of the Unexpected relative to the Expected condition.

To quantify the difference between the observed data and the estimated data, we compute the residual error: $y-{\hat{y}}$ (Fig. 8, right). We find that the residual error, averaged per time sample and participant and then split up by condition, is close to zero, indicating that the rERP models recover the effect structure of the observed data.

Strikingly, while the effect structure clearly differs across the three midline electrodes (e.g., compare the absence of a P600 effect at Fz to the presence of such an effect at Pz), the coefficient graphs look very similar. That is, at all electrodes, the coefficient for the N400 predictor suggests that more negative N400 amplitudes also induce more positive P600 amplitudes. While this may initially appear to be inconsistent, we will later address in detail how a monophasic effect structure can indeed yield the pattern of coefficients such as the one observed at Fz (see section “Single-trial dynamics in monophasic effect structures”).

In order to further decompose the extent to which specifically the N400 predictor—and not the Segment predictor—captures the condition contrast, we compute their isolated estimates and residuals. To do so, we use the models fitted with both predictors present and neutralise the influence of one of the predictors on the forward estimates, by setting the predictor values to their average, which is zero for z-standardised predictors. Crucially, this does not involve refitting the models, and thus the coefficients remain unchanged: That is, we only re-estimate data, using the same set of fitted coefficients, while neutralising different predictors.

**Fig. 9:**
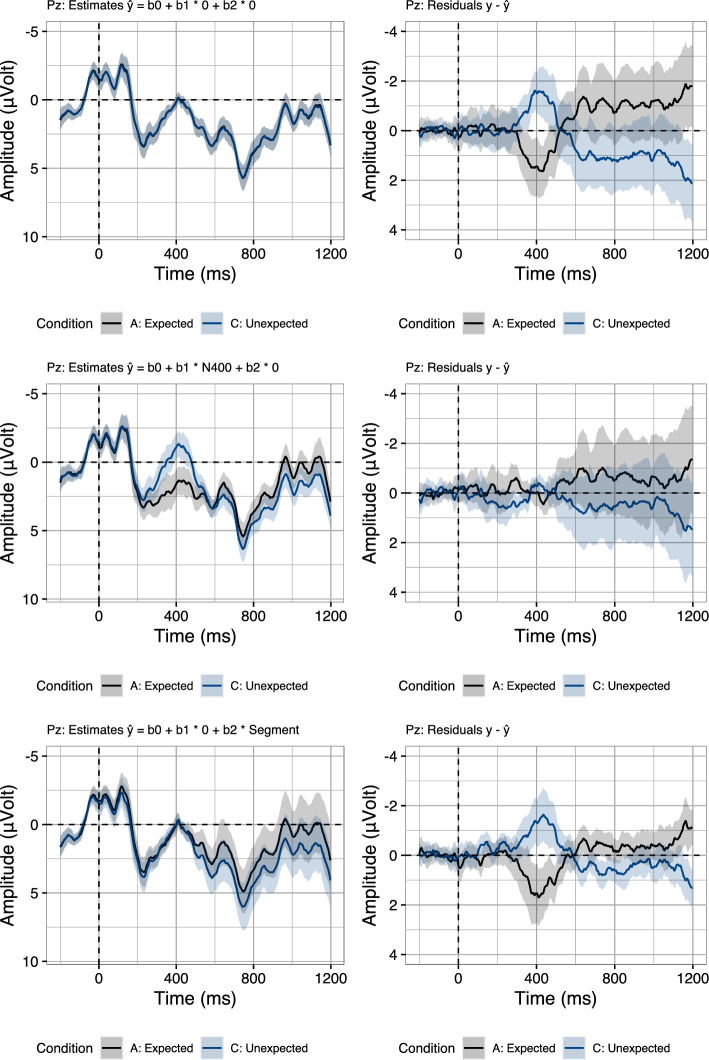
Isolated forward estimates (left) and residual error (right) computed from rERP models fitted with all predictors present (Eq. 1). Estimates and residuals were split per condition. Rows contain the isolated estimates and residuals of the intercept (row 1), the intercept plus the N400 predictor (row 2), and the intercept plus the Segment predictor (row 3). Error ribbons indicate confidence intervals based on standard errors computed across subjects

As the coefficients for the z-standardised predictors adjust the by-trial estimates in terms of their deviation from the grand average, as given by the intercept, a first step is to isolate the contribution of the intercept to the estimates by neutralising *both* the N400 and Segment predictor (see Fig. 9, row 1):

2 $$\begin{aligned} {\hat{y}}_{st} = \beta {0}_{st} + \beta {1}_{st} * 0 + \beta {2}_{st} * 0 \end{aligned}$$

Next, to compute the isolated estimates of the N400 predictor while neutralising the influence of the Segment predictor, we re-estimate the data using the following equation (see Fig. 9, row 2):

3 $$\begin{aligned} {\hat{y}}_{st} = \beta {0}_{st} + \beta {1}_{st} * \text {N400}_{st} + \beta {2}_{st} * 0 \end{aligned}$$

Conversely, to isolate the contribution of the Segment predictor, we neutralise the influence of the N400 predictor, as shown in the following equation (see Fig. 9, row 3):

4 $$\begin{aligned} {\hat{y}}_{st} = \beta {0}_{st} + \beta {1}_{st} * 0 + \beta {2}_{st} * \text {Segment}_{st} \end{aligned}$$

Plotting the isolated estimates and their residual error (Fig. 9), we first find that, trivially, the intercept, which is equal to the average of the data in our models, is a good model of the conditions pre-N400, but does not accurately capture the difference between conditions in the N400 and the P600 time-window (top row). Adding the N400 predictor to the computation of the forward estimates reveals that, indeed N400 amplitudes allow us to not only model the N400 effect, but also reduces the residuals in the P600 time-window, indicating that indeed, N400 amplitudes are predictive of P600 amplitudes (middle row). Lastly, turning to the Segment predictor, we find that, in fact, Segment voltage also models part of the P600 effect in the data (bottom row). This is most likely the case because the P600 is a long, sustained component and hence the Segment predictor also contains systematic P600 variability. Indeed, we find that single-trial P600 voltages (600–1000 ms) and single-trial Segment voltages (0–1200 ms) are strongly correlated (*r* = 0.94). Despite the fact that the P600 effect is in part modelled by Segment voltage, the isolated estimates of the N400 predictor (Fig. 9, middle row) reveal a unique contribution in explaining P600 variability, over and above what is accounted for by the Segment predictor.

#### Single-trial dynamics within-condition

**Fig. 10:**
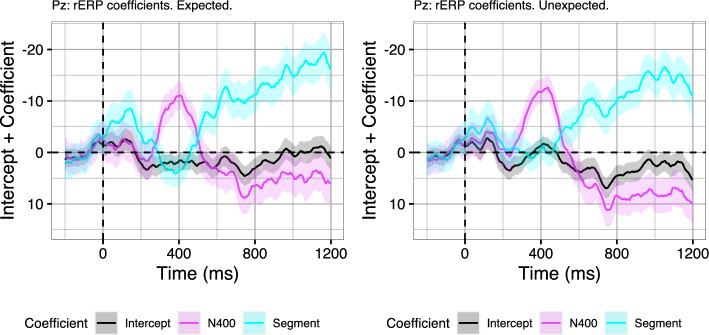
Model coefficients (added to their intercept) across time on electrode Pz for regression models fitted separately on the Expected and Unexpected condition of Aurnhammer et al. (2021). Coefficients express the extent to which single trial N400 amplitude (averaged from 300 to 500 ms) and Segment amplitude (averaged from 0 to 1200 ms) explain the EEG signal across time. Error ribbons indicate standard errors on the coefficients

While the estimates reveal that our rERP models successfully account for the ERPs at the condition level, it is an open question to what extent the observed N400–P600 interrelation is driven by the Unexpected and the Expected condition, respectively. Indeed, Aurnhammer et al. (2021) also conducted a post-hoc analysis that explored whether the graded expectancy of the target word in the Expected condition (Cloze probability: mean = 0.67, SD = 0.23, range = 0.17 - 1) also induced graded retrieval effort (N400) and integration effort (P600). An rERP analysis in which the EEG was modelled as a function of log-Cloze probability suggested that, indeed, not only N400 amplitude but also P600 amplitude was continuously related to target word expectancy. Hence, neither the N400 nor the P600 response appear to be specifically elicited by the violation of the main verb’s selectional restrictions that was employed in the Unexpected condition, which is in line with the assumption of RI theory that both components are continuous indices of processing effort. In the current analyses, we would thus expect that, similarly, the correlation of N400 amplitude and P600 amplitude should also be observable in the Expected condition alone and not driven by the Unexpected items alone. Thus, in order to validate that the N400–P600 interrelations we found are not qualitatively different in the Expected and the Unexpected condition, we also fit the rERP models for the two conditions separately, using the same regression equations as above. While it is now not possible to validate model fit against the effects observed between conditions (cf. Fig. 9), the model coefficients for the regressions that were fitted on the two conditions separately do not suggest any qualitative differences between the conditions on midline electrodes (see Fig. 10, for the coefficients at electrode Pz).

Hence, our novel approach reinforces that both the N400 (Kutas and Hillyard 1984) and the P600 (Aurnhammer et al. 2023) are continuous indices of processing effort, but importantly go beyond these earlier findings by also suggesting that the two ERP components are negatively correlated at the single-trial level both for well-formed sentence completions (Expected condition) and violating target words (Unexpected condition). However, while we found evidence for negatively correlated N400 and P600 amplitudes in a design that resulted in a biphasic effect between conditions, an open question is how these proposed within-trial dynamics can be reconciled with ERPs that exhibit only monophasic effects.

#### Single-trial dynamics in monophasic effect structures

While linguistic manipulations often elicit biphasic effects between conditions, there are many crucial ERP studies in which only an N400 effect or only a P600 effect was reported relative to baseline (due in part to spatiotemporal component overlap). Intuitively, it may seem that these monophasic *effects* would speak against or at least limit the N400–P600 within-trial dynamics predicted by RI theory. However, while experimental manipulations can be constructed such that retrieval effort is equal across conditions, leading to the absence of an N400 effect, or such that integration effort is equal across conditions, leading to the absence of a P600 effect, this is not necessarily at odds with the general proposal of RI theory that expectations derived from the utterance meaning representation constructed so far will modulate both retrieval and integration. That is, even in data in which no N400 or P600 effect is observed between conditions, a *within-condition* correlation between N400 amplitude and P600 amplitude may be present at the single-trial level.

**Fig. 11:**
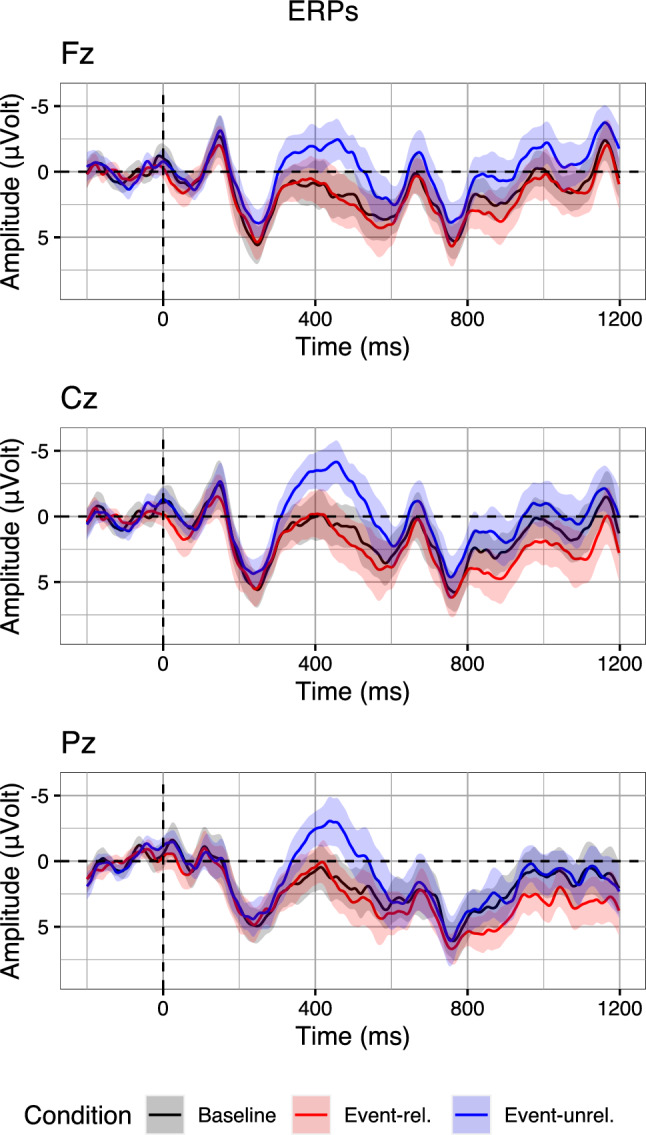
Average ERPs on three midline electrodes (Fz, Cz, Pz) in the baseline, event-related violation, and event-unrelated violation condition of Delogu et al. (2019). Error ribbons indicate confidence intervals based on standard errors computed across subjects

To illustrate this point, we turn to the data presented by Delogu et al. (2019) which, relative to baseline, revealed only a sustained N400 effect in one condition and only a P600 effect in another condition (but see Brouwer et al. 2021; Delogu et al. 2021). During the experiment, a context sentence was presented which introduced a scenario which was then followed by a critical sentence, presented word-by-word, containing the target word (see Table 2).^5^ The experiment consisted of three conditions in which the context sentence was either associated (“John entered/left the restaurant”) or unassociated (“John entered the apartment”) to the target word in the second sentence (“Before long he opened the menu and...”). Both manipulated conditions created a violation of world knowledge (opening the menu after leaving the restaurant or after entering the apartment). However, target word meaning in the *event-related* violation is associated to the context whereas it is unassociated in the *event-unrelated* violation. Delogu et al. (2019) found a P600 effect but no N400 effect for the event-related condition, relative to the baseline condition (Fig. 11). For the event-unrelated condition, an N400 effect but no P600 effect was found relative to the baseline.

**Table 2:** Example stimuli from Delogu et al. (2019)

Baseline	John entered the restaurant. Before long, he opened the menu and.
Event-related violation	John left the restaurant. Before long, he opened the menu and.
Event-unrelated violation	John entered the apartment. Before long, he opened the menu and.

Stimuli were transliterated from German. Context sentences were presented as a whole, critical sentences were presented using rapid serial visual presentation. Target words were underlined for this table

The finding that the event-related violation elicits no N400 effect and only a P600 effect relative to baseline is in line with RI theory: The context associatively facilitates target word retrieval similarly in both the baseline and the event-related violation condition, explaining the absence of an N400 effect. Integration, however, is more effortful in the event-related violation condition than in the baseline condition, leading to an increase in P600 amplitude. Importantly, for the event-unrelated condition, RI theory predicts a biphasic N400–P600 effect relative to control, as both retrieval and integration should be more effortful than in baseline condition. However, only an N400 effect and no P600 effect was observed. The absence of the predicted P600 *effect* in the event-unrelated condition relative to baseline is explainable in terms of spatiotemporal component overlap (Luck 2005) between the N400 and the P600 component (see Brouwer et al. 2021, for evidence and Brouwer and Crocker 2017, for a general discussion). The N400 and the P600, being opposite in polarity, partly cancel each other out in the scalp recorded signal, which may result in the attenuation—or even absence—of a P600 *effect* between conditions in the observed data. Indeed, in a follow up study, the N400 effect disappears and the P600 effect re-emerges if the event-unrelated condition is compared to a similarly unassociated baseline condition (Delogu et al. 2021). Critically, while component overlap can lead to puzzling effects structures, this is not necessarily a problem for analyses of single-trial data: For instance, in the contrast of the event-unrelated condition to the baseline condition, average P600 amplitude may be equal in both conditions, which may seem difficult to reconcile with the large difference in average N400 amplitude when assuming correlated N400 and P600 amplitudes. However, while average N400 amplitudes may be offset in the two conditions, there may still be a correlation between ERP components within-condition.

**Fig. 12:**
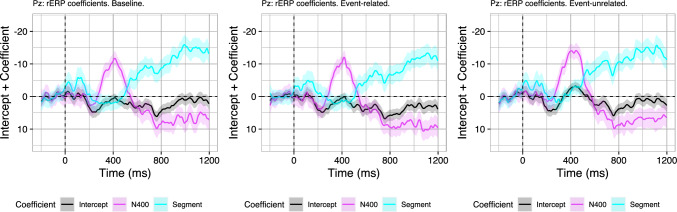
Model coefficients (added to their intercept) across time on electrode Pz for regression models fitted separately on the experimental conditions of Delogu et al. (2019). Coefficients express the extent to which single trial N400 amplitude (averaged from 300 to 500 ms) and Segment amplitude (averaged from 0 to 1200 ms) explain the EEG signal across time. Error ribbons indicate standard errors on the coefficients

In order to determine the single-trial dynamics in the Delogu et al. (2019) data, we conduct our analyses separately in the three conditions (analogous to Sect. Single-trial dynamics within-condition). Again, this means that we cannot rely on evaluating the regression models against the effects structure observed between conditions. While we previously quantified the extent to which the regression models capture the P600 effect between conditions, there is no P600 effect for the contrast of the event-unrelated condition relative to baseline (due in part to spatiotemporal component overlap; Delogu et al. 2021; Brouwer et al. 2021). Similarly, while there is a P600 effect for the event-related condition relative to baseline, here the average N400 amplitudes—and hence the N400 predictor values in the regression models—do not differ across conditions. Hence, assessing the extent to which the rERP models capture the effect-structure across conditions is not informative. As before, however, the regression coefficients are still informative. Fitting rERP models separately for each condition, we find similar patterns as before (Fig. 12 shows the coefficients at Pz). Indeed, in each of the three analyses, the intercept term is equal to the average of the condition. The coefficient of the N400 predictor suggests, as before, that the variability around the mean is correlated in the N400 and the P600 time-window: Within each of the three condition of Delogu et al. (2019), more negative N400 amplitudes co-occur with more positive P600 amplitudes within-trial. In sum, our analyses do suggest that while the average N400s and average P600s may or may not differ between conditions, there is a within-trials correlation between the two ERP components within-condition. Indeed, these findings are also consistent with the computational model instantiation of RI theory (Brouwer et al. 2021) that generated N400 and P600 estimates for the items in the Delogu et al. (2019) study. Within-condition, we find that the N400 and P600 amplitudes predicted by the computational model instantiation for the target words are negatively correlated (Baseline: $r = -0.62$; Event-related violation: $r = -0.52$; Event-unrelated violation: $r = -0.50$).

## Discussion

Ultimately, any viable model of the neurocognition of language comprehension should explain how the N400 component and P600 component of the ERP signal are modulated at the single trial-level. While most computational instantiations of neurocognitive models do indeed make such trial-level predictions, the statistical analysis and interpretation of N400 and P600 modulations in ERP data is often focused on the effect level, comparing condition averages in pre-defined time-windows. We have argued that this focus on effects limits our ability to decide between models, and that we may improve upon this situation by moving from the effect level to the level of single trials.

We demonstrate this approach by teasing apart two explanations of biphasic N400–P600 effect patterns: On Multi-stream accounts, the N400 increases and the P600 increases are thought to stem from different pools of trials. That is, certain trials elicited by semantic anomalies that induce an N400 increase should not induce a P600 increase, whereas other trials that do not induce an N400 increase should induce a P600 increase. On RI theory, on the other hand, trials with more negative N400 amplitudes are predicted to also exhibit more positive P600 amplitudes. Critically, a set of regression models with single-trial N400 averages as predictor is able to explain systematic variability in the P600 time-window and recovers the effects structure observed for the expectancy manipulation by Aurnhammer et al. (2021). Further, an analysis of the Expected condition in isolation suggests that this relation is not specific to target words that violate selectional restrictions of the verb (Unexpected condition: “Then ate the lumberjack the axe”). This forms strong support for the explanation of RI theory, and demonstrates that N400 amplitudes and P600 amplitudes are correlated at the single trial level. Importantly, we also demonstrate that the predicted correlation between N400 and P600 amplitude is not generally at odds with monophasic effect patterns, as we found similar N400–P600 couplings within the individual conditions of Delogu et al. (2019). The key to this explanation are differences in per-condition average N400 or P600 amplitude, which may, for instance, be induced by strong priming or spatiotemporal component overlap. In sum, our results are in line with RI theory and not only eschew the need for multi-stream architectures but present explicit counter-evidence for the single-trial dynamics that follow from multi-stream architectures.

In contrast to these multi-stream models, RI theory posits a single-stream architecture in which expectation-based language comprehension is driven by an utterance meaning representation that is updated with every incoming word. During processing of a word, the utterance meaning representation constructed so far influences both the mapping of word forms to word meaning representations (retrieval/N400) and the updating of the utterance meaning representation with the retrieved word meaning (integration/P600). Due to the strong influence exerted by the utterance meaning representation on both retrieval and integration, N400 amplitude and P600 amplitude are predicted to be inversely correlated: Words that require more effort to retrieve, will generally be more effortful to integrate, and, consequently, more negative N400 amplitudes should, generally, co-occur with more positive P600 amplitudes. Note that the relationship of N400 amplitude and P600 amplitude is strictly *correlational*. That is, beyond the effects of spatiotemporal component overlap (see Brouwer and Crocker 2017, for discussion; also see Brouwer et al. 2021), there is no direct *causal* relationship between *latent* N400 amplitude and P600 amplitude in the signal itself. Rather, on RI theory, there is a causal relation between both the retrieval process underlying the N400 and the integration processes underlying the P600 to the utterance meaning representation constructed so far. It is *this* mechanistic dependence of both retrieval and integration on the unfolding utterance representation that underlies the observed correlation in the signal itself.

As a consequence of this architecture, RI theory assumes that both the N400 and the P600 component are elicited by every word during language comprehension. Hence, biphasic N400–P600 patterns should be considered to be part of the default ERP signature of language processing. Crucially, this proposal is not at odds with the absence of N400 or P600 *effects* in certain condition contrasts. Rather, monophasic *effects* would be explained through conditions consisting of stimuli that are matched in the degree to which they make retrieval (N400) or integration (P600) effortful. Further, spatiotemporal component overlap between the N400 and the P600 can result in the partial cancellation of ERP components, which can render the observed condition-averaged waveforms unrepresentative of the underlying latent components (Brouwer and Crocker 2017; Brouwer et al. 2021; Delogu et al. 2021).

Additionally, the neurocomputational RI model directly predicts continuous N400 and P600 amplitude modulations, rather than binary increase patterns. This is critical, since the N400 component has been shown to be a graded processing index (Kutas et al. 1984) and a similar gradedness has recently been demonstrated for the P600 (Aurnhammer et al. 2023; see also the post-hoc analyses in Aurnhammer et al. 2021). Hence, models of the electrophysiology of language comprehension should aim to generate continuous estimates of processing cost that reflect the graded nature of ERPs.

Lastly, it is worth noting that our single-trial analysis also goes beyond the item level: Both model-derived and human-derived processing estimates (such as Cloze probability) are computed for stimuli, abstracting over the notion of individual participants, who may experience variable processing effort. Our analyses are, however, informed by single-trial N400 amplitudes and suggest that even at this level of granularity, N400 and P600 amplitude are correlated. We interpret this as converging evidence for previous studies demonstrating that individual participants’ understanding and knowledge drives expectation-based language comprehension (Troyer and Kutas 2020; Troyer et al. 2020).

## Conclusion

Most theories of the electrophysiology of language comprehension are informed by, and make predictions about ERP effects between conditions. There are multiple shortcomings with this approach: Focusing on effects bears the risk of artificially dichotomising the demonstrably continuous sensitivities of the N400 and the P600 and hence may obscure crucial aspects of EEG data that could inform theories. Further, spatiotemporal component overlap between the N400 and the P600 may result in a divergence between the observed ERP effects and the underlying, latent component structure. Finally, competing accounts for a range of ERP data at the effect-level assume fundamentally different language processing architectures. Here, we addressed these shortcomings by examining ERP data at the single-trial level. To do so, we articulated trial-level predictions of competing theories—multi-stream models and RI theory—for biphasic N400–P600 patterns observed between conditions. We then investigated the within-trial dynamics of the N400 and the P600 component. Using a regression-based approach, we quantified the extent to which single-trial N400 amplitudes are predictive of their consecutive P600 amplitudes. We provide first evidence that their amplitudes are continuously and inversely correlated: Trials with larger N400 amplitudes also exhibit larger P600 amplitudes. Further, we have shown that this finding is not limited to biphasic effect patterns, but also extends to monophasic effect patterns.

The finding that increases in N400 and P600 amplitude are coupled within-trial supports the single-stream view proposed by Retrieval-Integration theory and appears inconsistent with the processing architecture proposed by many Multi-stream models, which predicts that any given trial should elicit either an N400 or a P600 increase. Our results illustrate that in order to further dissociate competing theories of the electrophysiology of language comprehension, models should make quantitative single-trial level predictions and, crucially, ERP analyses must evaluate these predictions at the trial level, rather than at the effect-level.

## Data Availability

All data and code required to reproduce the analyses is publicly available at https://github.com/caurnhammer/cody23rerps. The data on which this research is based (Aurnhammer et al. 2021; Delogu et al. 2019) has been obtained with ethics approval of the Deutsche Gesellschaft für Sprachwissenschaft (DGfS). All participants gave informed consent in written form.

## Footnotes

1 While there are numerous other theories about the N400 component and the P600 component in isolation (see Delogu et al. 2019, for discussion), we here seek to investigate the single-trial dynamics of the N400 and the P600, and therefore focus on *integrated* theories of the N400 and the P600 only.

2 2 https://github.com/caurnhammer/cody23rerps.

3 Computed using the ppcor package for R (Kim 2015).

4 Statistical analyses were implemented in Julia (Bezanson et al. 2017).

5 EEG data were processed the same way as described previously for Aurnhammer et al. (2021) in Sect. Method. For full detail on experimental design, electrophysiological recording and processing, see Delogu et al. (2019).
